# Host Lipid Rafts as the Gates for *Listeria monocytogenes* Infection: A Mini-Review

**DOI:** 10.3389/fimmu.2020.01666

**Published:** 2020-08-11

**Authors:** Yu-Huan Tsai, Wei-Lin Chen

**Affiliations:** Laboratory of Host–Microbe Interactions and Cell Dynamics, Institute of Microbiology and Immunology, National Yang-Ming University, Taipei, Taiwan

**Keywords:** *Listeria monocytogenes*, listeriosis, lipid rafts, intracellular bacteria, listeriolysin O, internalin, cell-to-cell spreading

## Abstract

*Listeria monocytogenes* is a Gram-positive foodborne bacterial pathogen capable of interacting and crossing the intestinal barrier, blood–brain barrier, and placental barrier to cause deadly infection with high mortality. *L. monocytogenes* is an intracellular pathogen characterized by its ability to enter non-phagocytic cells. Expression of the cytolysin listeriolysin O has been shown to be the main virulence determinant *in vitro* and *in vivo* in mouse models. *L. monocytogenes* can also perform cell-to-cell spreading using actin-rich membrane protrusions to infect neighboring cells, which also constitutes an important strategy for infection. These events including entry into host cells, interaction between listeriolysin O and host plasma membrane, and bacterial cell-to-cell spreading have been demonstrated to implicate the cholesterol-rich lipid rafts or molecules in these microdomains in the host plasma membrane *in vitro* with tissue culture models. Here we review the contribution of lipid rafts on plasma membrane to *L. monocytogenes* infection.

## Introduction

Human listeriosis is a foodborne disease caused by the intracellular pathogen *Listeria monocytogenes*. Upon ingestion of contaminated food by the host, *L. monocytogenes* interacts and traverses the intestinal epithelium to reach the lamina propria, followed by dissemination into lymph and bloodstream toward the liver and spleen, where the bacteria replicate. *L. monocytogenes* can further cross the blood–brain barrier to induce meningoencephalitis and invade the placenta and result in fetal infection, stillbirth, abortion, and neonatal infection (1, 2). *L. monocytogenes* has been used as a model microorganism in studying host–microbe interactions since the late 1980s. Efforts have been made to unveil how this facultative intracellular pathogen enters into cultured non-phagocytic epithelial cells, escapes from the internalization vacuole, and spreads from the infected cell to another (3). Interaction of *L. monocytogenes* surface protein InlA and InlB with corresponding host receptors human E-cadherin (hEcad) and human c-Met at the plasma membrane leads to host actin polymerization and septin assembly, followed by internalization of bacteria in a vacuole (4–7). The *L. monocytogenes*-containing vacuole, especially in phagocytic cells, is subsequently lysed by the bacterial pore-forming toxin listeriolysin O (LLO), resulting in bacterial escape from the vacuole (8). Within the host cell cytosol, the bacterial surface protein ActA contributes to *L. monocytogenes* intracellular motility via host actin polymerization and actin comet tail formation, followed by the induction of membrane protrusions and infection of neighboring cells (9). In the neighboring cells, *L. monocytogenes* will then be located in a double-membrane vacuole, in which bacterial phospholipases PlcA and PlcB, together with LLO, are required for vacuole lysis and bacterial escape into cytosol for infection propagation (10). The membrane damaging property of LLO is also involved in multiple modulation activities during infection, such as Arp2/3-dependent F-actin remodeling that promotes bacterial internalization, changes in histone modification and thus modulation of host gene expression, desumoylation of host proteins, induction of mitochondrial fission, increase of endoplasmic reticulum (ER) stress, and lysosomal permeabilization (11–19).

**Figure 1:**
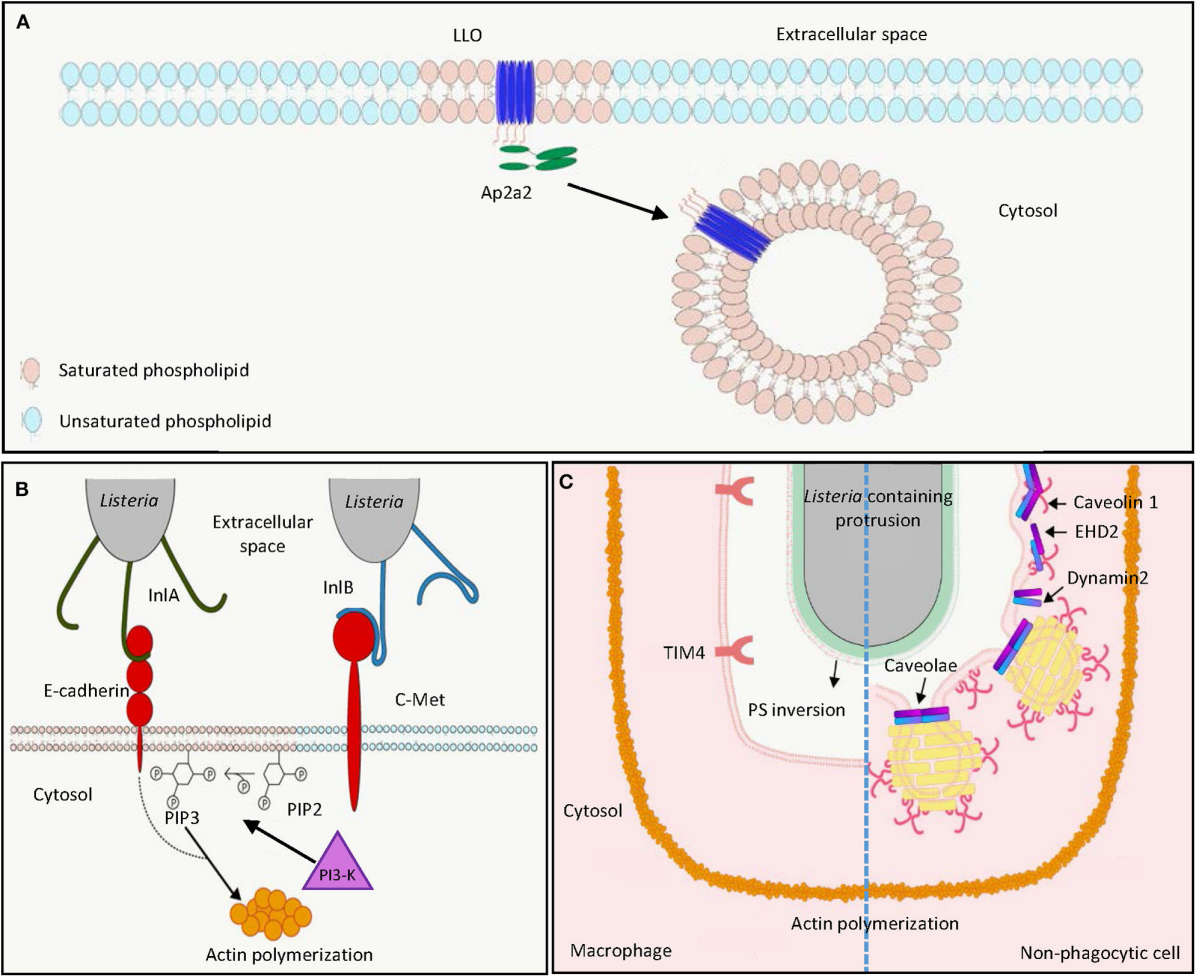
*L. monocytogenes* harnesses host lipid rafts for infection. **(A)** Extracellular *L. monocytogenes* secretes the cytolysin LLO, which binds cholesterol and inserts into host cell membrane in lipid raft domains. The interaction between the endocytosis adaptor protein Ap2a2 and inserted LLO results in endocytosis of LLO to prevent LLO-induced plasma membrane damage. **(B)** During bacterial entry, the *L. monocytogenes* surface protein InlA acts as an adhesin binding to host E-cadherin in lipid raft domains. Binding of *L. monocytogenes* InlB to host c-Met triggers PI3-K activation, which catalyzes the production of PIP3 from PIP2 in lipid raft domains, leading to actin polymerization and internalization of E-cadherin-bound bacteria. **(C)** In the process of cell-to-cell spreading, LLO damages the plasma membrane of the infected cells to induce PS inversion in the lipid rafts. Neighboring macrophages engulf PS-positive protrusion structures by the PS receptor TIM4. Neighboring non-phagocytic cells internalize *L. monocytogenes* membrane protrusions by caveolin-dependent endocytosis in lipid rafts. LLO, listeriolysin O; PIP2, phosphatidylinositol-4,5-bisphosphate; PIP3, phosphatidylinositol-3,4,5-triphosphate; PI3-K, phosphoinositide 3-kinase; PS, phosphatidylserine.

Plasma membranes have been described as a fluid mosaic interface containing various lipid species in two asymmetric leaflets with plenty of floating proteins (20, 21). The observations that cell membranes can be separated into detergent-sensitive and detergent-resistant fractions suggested the presence of distinct membrane sub-compartments in cell membranes. The clusters of lipids in a more ordered state with relatively saturated lipids and glycosylated lipids are referred to as lipid rafts, as compared to the disordered membrane domains with unsaturated lipids (20). Lipid rafts are cholesterol- and sphingolipid-enriched microdomains with ordered assemblies of proteins and lipids in cell membranes (22). These rafts are heterogeneous and dynamic, and have the potential to form large domains (>300 nm) upon clustering induced by protein–protein and protein–lipid interactions (20). The organization of lipid rafts can also be regulated and mobilized by cortical actin filaments via specific interactions between actin and membrane adaptor proteins (23, 24). Lipid rafts have been suggested to be implicated in membrane protein signaling, membrane trafficking, and host–microbe interactions (25). Compartmentalization of cellular signaling in membrane domains is important to regulate maturation of immune cells. It was demonstrated that T cell receptors and B cell receptors were found in detergent-sensitive membrane in resting stage, but shifted to detergent-resistant fractions upon receptor activation (26–29). This suggests that the translocation of receptors involved in antigen presentation to lipid rafts is associated with active signaling in these immune cells. Lipid rafts are also enriched in caveolae, which are flask-shaped pits with a size of 50–80 nm in the cell membrane. Caveolae are associated with expression of caveolin, which is responsible for stabilization of caveolar structure and the internalization of extracellular materials into caveolae (25). While host plasma membranes are the first barrier for the invasion of intracellular pathogens, the observation that host receptors are clustered in the lipid rafts and enrichment of cholesterol on microbe-containing vacuoles highlights the importance of lipid rafts in pathogen–host interactions (30). Here we review and discuss the implication of lipid rafts in the interaction between *L. monocytogenes* and host cells at different interfaces (Figure 1).

## LLO, a Multifunctional Cytolysin Targeting Lipid Rafts

LLO is crucial for full virulence of *L. monocytogenes* in both *in vitro* tissue culture systems and *in vivo* animal models (31–35). LLO is a secreted protein of 56 kDa molecular weight belonging to the family of cholesterol-dependent cytolysins (CDCs), which represent the largest family of pore-forming toxins that form large pores (up to 35 nm) produced by different bacterial species (36–40). Preincubation of LLO with cholesterol abolished cytolytic activity, suggesting the importance of cholesterol binding in lipid rafts for cytolysis (41). Differing from other CDC members, LLO exhibits optimal binding to cholesterol-containing membranes at pH 5.5, and this binding decreases at neutral and basic pH. Nevertheless, high cholesterol levels, corresponding to the concentration range of cholesterol found in lipid rafts, can restore LLO binding to membranes at suboptimal pH (42). This is explained by the presence of an acidic triad in the transmembrane domain, which functions as a pH sensor and triggers premature denaturation of LLO at neutral pH at 37°C, thereby allowing pore formation to occur mainly at acidic pH (43–45). The pH dependence of LLO limits cytolytic activity to acidic vesicles and prevents damage in the host cytosol, which is a niche for *L. monocytogenes* replication. Accordingly, replacement of LLO by pH-insensitive CDCs such as perfringolysin O from *Clostridium perfringens* allowed phagosomal escape of *L. monocytogenes*, but led to decreased infection efficiency *in vitro* in a plaque assay (46). Cholesterol in the lipid rafts not only provides an initial binding site for LLO, *in vitro* studies with high-speed atomic force microscopy (HS-AFM) further demonstrated that in acidic environments LLO can produce arc pores in the membrane as a lineactant, and therefore creates large-scale defects for bacterial escape from phagocytic vacuole (47).

In addition to targeting of cholesterol-rich domains to damage the host cell membrane, the PEST-like sequence at the N-terminus of LLO interacts with the endocytosis adaptor Ap2a2, a lipid-raft associated protein (Figure 1A) (48, 49). This interaction facilitates clathrin-dependent endocytosis of plasma membrane-associated LLO and removes these pore-forming toxins from the plasma membrane, thereby preventing its cytotoxicity to the infected cell and enhancing *L. monocytogenes* virulence during infection *in vivo* (48, 50). However, this clathrin-dependent endocytosis of LLO-associated membrane may not contribute to membrane repair after pore formation as endocytic proteins are not recruited to the membrane damage sites (51). Instead, LLO-induced membrane damage can result in influx of intracellular calcium, which subsequently activates TMEM16F lipid scramblase, leading to membrane blebbing and extracellular vesicle release to repair plasma membrane damage (52). Although less characterized, LLO was demonstrated to induce clustering of GPI-anchored proteins CD14 and CD24 on the surface of murine macrophages J774, while the non-lipid-raft marker transferrin receptor was not affected (53).

## *L. monocytogenes* Harnesses Lipid Rafts Proteins for Entry Into Host Cells

*L. monocytogenes* uses its surface internalin proteins InlA and InlB to bind hEcad and human c-Met, respectively. These interactions result in cytoskeleton rearrangement, thereby bacterial entry into non-phagocytic cells through a zipper mechanism (Figure 1B) (4, 7, 54). Treatment of phosphoinositide 3-kinase (PI3-K) inhibitors wortmannin and LY294002, respectively, abolished InlA- and InlB-dependent invasion into different host cells, showing the importance of PI3-K activity in internalin-mediated entry (Figure 1B) (55, 56). Depletion of cholesterol by methyl-β-cyclodextrin reduced InlA- and InlB-dependent *L. monocytogenes* internalization into non-phagocytic cells, indicating the involvement of lipid rafts in internalin-mediated internalization (57). This is further supported by the observation that multiple lipid raft markers, such as glycosylphosphatidylinositol-linked proteins, a myristoylated and palmitoylated peptide, and the ganglioside GM1 were recruited at the bacterial entry site (57). While InlA–Ecad interaction and Ecad recruitment at the entry site were cholesterol-dependent, cholesterol depletion did not affect InlB interaction with c-Met, the recruitment of c-Met at the entry site, and c-Met downstream signaling (57). Nevertheless, cholesterol depletion abrogates InlB-mediated actin polymerization (57). The implication of lipid rafts in InlA-mediated entry was further demonstrated by the observation that caveolin was recruited to the bacterial entry sites and was required for the internalization in an InlA-dependent manner (58). While InlB is dispensable for bacterial entry into LS174T intestinal epithelial cells that show constitutively activated PI3-K activity, this protein is necessary for InlA-dependent entry into cells that do not exhibit constitutive PI3-K activity (55). Together, while InlB-induced c-Met phosphorylation does not depend on cholesterol and lipid rafts, subsequent PI3-K activation and Rac1-induced actin polymerization at the bacterial entry site occur within lipid rafts and require their integrity (57, 59). InlA acts as an adhesion molecule to Ecad in the detergent-resistant lipid rafts, thereby triggering internalization of *L. monocytogenes* dependent on PI3-K activation and caveolin-mediated endocytosis (Figure 1B) (57–60).

## The Role of Lipid Rafts in *L. monocytogenes* Cell-to-Cell Spreading

Following lysis of the phagocytic vacuole in host cells, the *L. monocytogenes* surface protein ActA can polymerize actin in cytosol, which allows formation of bacteria-containing membrane protrusions, the structures that are later internalized by neighboring cells to result in cell-to-cell spreading of *L. monocytogenes* (Figure 1C) (61). Internalization of *L. monocytogenes* membrane protrusions was demonstrated to be exploited by efferocytosis, by which the apoptotic cells are removed by macrophages (Figure 1C, left) (62). In both phagocytic and non-phagocytic cells, ActA-mediated actin-based motility was proposed to allow close apposition of bacteria to the cell membrane, where secreted LLO may damage cell membrane and induce externalization of phosphatidylserine (PS), a hallmark of apoptotic cells at cell membrane (62). The PS-positive protrusion structures containing *L. monocytogenes* are subsequently recognized by the PS-binding receptor TIM-4 on macrophages to facilitate phagocytic uptake and cell–cell spreading (62). While methyl-β-cyclodextrin-mediated cholesterol depletion was demonstrated to reduce PS externalization and phagocytosis of apoptotic cells, this suggests that lipid raft integrity could be important for efferocytosis-mediated *L. monocytogenes* spreading (63–65). *L. monocytogenes* cell-to-cell spreading is not limited to transfer to phagocytic cells (61). Dhanda et al. further investigated the membrane invagination in neighboring non-phagocytic cells (Figure 1C, right) (66). While caveolin-based and lipid raft-dependent endocytosis is supposed to be a process that internalizes extracellular material into bulb-shaped caveolae no larger than 100 nm, *L. monocytogenes* membrane protrusions triggered the recruitment of caveolar proteins and PS in a neighboring cell (66). Knock-down of caveolin-1 reduced invagination length in the neighboring cells and *L. monocytogenes* cell-to-cell spreading without detectable effect on the length of the actin comet tail and protrusion in initial infected cells (66). This suggests that caveolin can mediate engulfment of large materials, such as *L. monocytogenes*-containing membrane protrusions, which was not supposed to be achieved based on the size of caveolae. Collectively, due to the importance of lipid rafts in both efferocytosis and caveolae structure, efficient *L. monocytogenes* cell-to-cell spreading may require lipid raft integrity (63, 67). Further studies disrupting these membrane microdomains in *L. monocytogenes* cell-to-cell spreading are needed to directly address the role of lipid rafts in this process.

## Concluding Remarks

Our understanding of lipid rafts has been improved by the development of biochemical and microscopy tools (20, 21, 25). Depletion of membrane cholesterol by methyl-β-cyclodextrin constitutes the most common approach to disrupt membrane lipid rafts to investigate their biological function (68). However, methyl-β-cyclodextrin exhibits pleiotropic effects beyond lipid raft disruption such as inhibition of clathrin-mediated endocytosis (69–71). The models where lipid raft components are genetically deficient may be applied to more specifically address the function of lipid rafts (72–74). On the other hand, advance in microscopy techniques allows for visualizing the lipid rafts from model membranes to living cells *in vitro* (25). Recent advances suggest that pathogens may behave differently and adopt a distinct strategy to interact with the host between *in vitro* cell culture and *in vivo* animal models. For example, while *L. monocytogenes* interacts with enterocytes and access to the cytosol in cell culture systems, it targets goblet cells in the small intestine and performs transcytosis to cross intestinal epithelium followed by systemic infection in humanized mouse models (55, 75, 76). Conditional genetic knock-out mouse models, where lipid rafts are disrupted in villin-expressing intestinal epithelium, may provide a clue regarding the role of lipid rafts in *L. monocytogenes* crossing of intestinal barrier (77). Stem cell-derived organoids have been shown to recapitulate the complexity of a local tissue *in vitro* in culture systems (78). While *L. monocytogenes* was demonstrated to successfully infect intestinal organoids, coupling of super-resolution optical microscopy methods may allow visualizing the organization of lipid rafts at the interface between *L. monocytogenes* and host cells relevant to the *in vivo* environment (79, 80). Together, future studies addressing the role of lipid rafts *in vivo* and visualizing these nanoscale membrane domains at the interface between *L. monocytogenes* and host in tissues will provide insight into how lipid rafts are implicated in the pathophysiology of *L. monocytogenes* infection.

## Author Contributions

Y-HT: conceived and designed the study, and wrote and edited the paper. W-LC: reviewed the literature and edited the paper. All authors contributed to the article and approved the submitted version.

## Conflict of Interest

The authors declare that the research was conducted in the absence of any commercial or financial relationships that could be construed as a potential conflict of interest.
